# Visuo-tactile feedback policies for terminal assembly facilitated by reinforcement learning

**DOI:** 10.3389/frobt.2025.1660244

**Published:** 2025-10-22

**Authors:** Yuchao Li, Ziqi Jin, Jin Liu, Daolin Ma

**Affiliations:** 1 School of Ocean and Civil Engineering, Shanghai Jiao Tong University, Shanghai, China; 2 School of Mechanical Engineering, Shanghai Jiao Tong University, Shanghai, China

**Keywords:** visual perception, tactile sensing, multi-modal fusion, terminal assembly, reinforcement learning

## Abstract

Industrial terminal assembly tasks are often repetitive and involve handling components with tight tolerances that are susceptible to damage. Learning an effective terminal assembly policy in real-world is challenging, as collisions between parts and the environment can lead to slippage or part breakage. In this paper, we propose a safe reinforcement learning approach to develop a visuo-tactile assembly policy that is robust to variations in grasp poses. Our method minimizes collisions between the terminal head and terminal base by decomposing the assembly task into three distinct phases. In the first *grasp* phase,a vision-guided model is trained to pick the terminal head from an initial bin. In the second *align* phase, a tactile-based grasp pose estimation model is employed to align the terminal head with the terminal base. In the final *assembly* phase, a visuo-tactile policy is learned to precisely insert the terminal head into the terminal base. To ensure safe training, the robot leverages human demonstrations and interventions. Experimental results on PLC terminal assembly demonstrate that the proposed method achieves 100% successful insertions across 100 different initial end-effector and grasp poses, while imitation learning and online-RL policy yield only 9% and 0%.

## 1 Introduction

Terminal assembly (McKee et al., 1985) is a precision manipulation task that involves part-to-part contact. Its four key sub-tasks—part feeding, object reorientation, peg insertion, and terminal buckling—have been widely investigated (McKee et al., 1985; Goldberg, 1993; Lozano-Pérez, 1986; Lozano-Perez et al., 1984; Natarajan, 1989). Early research primarily focused on mechanical design aspects (Lozano-Pérez, 1986; Natarajan, 1989) and motion planning strategies (Goldberg, 1993; Lozano-Perez et al., 1984; Qiao et al., 1995). With the aid of Computer-Aided Design (CAD), the assembly sequence can be pre-defined in simulation using accurate pose information (De Mello and Sanderson, 1989), enabling robots to plan the required actions for executing the assembly (Koga et al., 2022). Recently, reinforcement learning (RL)-based approaches have demonstrated potential in handling assembly tasks involving parts with complex geometries (Wen et al., 2022; Lian et al., 2021). However, RL remains challenging due to the requirement for frequent human inputs during learning (Luo et al., 2021) or high-precision sensors for collecting training data (Wen et al., 2022). Meanwhile, because the terminal head has the characteristics of irregular shape, easy damage, there is also a need for a safe training and data collection method for learning assembly tasks.

Another challenge in terminal assembly tasks is that the precise initial pose of the terminal is often unknown. Since the grasped object is frequently visually occluded by the gripper, tactile sensing provides a more effective means for grasp pose estimation (Okumura et al., 2022; Dang et al., 2023). Although recent advances have demonstrated improved simulation accuracy for industrial insertion tasks (Narang et al., 2022), and successful Sim2Real transfer has been achieved for tactile-based insertion tasks (Kelestemur et al., 2022; Wang et al., 2022), simulating soft contacts between tactile sensors and objects with complex geometries remains an open challenge (Wang et al., 2021). This issue often hinders real-world transfer, as accurate object models are rarely publicly available. Additionally, a major obstacle in applying reinforcement learning (RL) to real-world terminal assembly tasks involving tactile feedback is the frequent slippage of parts caused by environmental collisions and the inherently smooth surface of the tactile sensor’s gel pad. Such slippage makes it difficult for RL methods to succeed without human intervention or the use of a dedicated pose estimation algorithm to detect and correct misalignments.

In this work, we present a novel method to safely learn visuo-tactile feedback policies in real for terminal assembly tasks under grasp pose uncertainties, with inexpensive off-the-shelf sensors. Our approach draws on tactile and visual feedback to deal with the uncertainty of grasp pose and a safe RL training procedure, minimizing damage during the training phase. We use Sample-Efficient Robotic reinforcement Learning (SERL) (Luo et al., 2024), a software suite that provides a well-designed foundation for robotic RL, to develop a data collection and training pipeline that minimizes collision between the part and its environment.

The whole pipeline can be divided into three steps: First, Training Reward Classifier: Labeling visual and tactile images from human instruction instances to train a reward classifier to decide when to give policy rewards throughout the RL training process. Second, Recording Demonstrations: To accelerate training, record a predetermined number of human-operated robot demonstrations to finish terminal assembly. This will serve as a demo buffer for RL. Third, Policy Training: Using the trained reward classifier and recorded demonstrations to complete the task training (during training, human interventions can be added to avoid collisions and speed up the training).

The main contributions of this paper are as follows: the development of a policy for complex terminal assembly in real-world scenarios, which leverages visual and tactile information through reinforcement learning and can be acquired in less than 60 min; the introduction of a safe exploratory strategy for reinforcement learning, accompanied by a secure data collection methodology grounded in a designated manual remote operation technique; and the presentation of experimental findings that indicate the policy attains a success rate of 100 out of 100 in Programmable Logic Controller (PLC) terminal assembly, thereby surpassing two baseline approaches that recorded success rates of 0 out of 100 and nine out of 100, respectively.

## 2 Related work

For many years, terminal assembly has been an essential part of robotics. The parts’ fragility, the moderate force during terminal buckling, the occlusions caused by the robot gripper, the grasp uncertainty from the acquisition process and its collision with the environment, and the precision required to control the robot for insertion render the task challenging. Early work approached this problem using CAD information to infer desired assembly sequences (De Mello and Sanderson, 1989) and generating designs of part feeders based on object geometry (Natarajan, 1989). Other work approached the problem from an algorithmic design perspective, with a focus on developing motion planning strategies for peg insertion (Lozano-Pérez, 1986; Qiao et al., 1995).

Recently, learning-based methods have shown success on this task. This includes learning assembly policies with a physical robot via Sim2Real transfer (Johannink et al., 2019), online adaptation with meta-learning (Schoettler et al., 2020b; Zhao et al., 2022), reinforcement learning (Luo et al., 2021; Schoettler et al., 2020a), self-supervised data collection with impedance control (Spector and Di Castro, 2021), accurate state estimation (Wen et al., 2022), or decomposing the assembly algorithm into a residual policy that relies on conventional feedback control (Johannink et al., 2019). These approaches assume that the parts are grasped with a fixed pose. To overcome this assumption, Wen et al. (Wen et al., 2022) perform accurate pose estimation and motion tracking with a high-precision depth camera and use a behavioral cloning algorithm to insert the part. Spector et al. (Spector and Di Castro, 2021; Spector et al., 2022) proposed Insertionnet for industrial assembly, which requires contact between the part and the environment to occur during data collection, a process that is expensive and often impractical for fragile parts. Ozalp et al. (2024) made advancements in deep RL and inverse RL for robotic manipulation. In comparison, we use inexpensive tactile sensors and a safe human-guided data collection and RL procedure that does not require such contact.

In systems using only visual perception, grasped parts are often visually occluded by the gripper, and changes in environment light can affect the accuracy of visual recognition. However, tactile perception is not affected by these factors: the camera of the tactile sensor is placed inside the body, so the collected tactile images will not be blocked by itself or environmental objects; the light source for tactile images is a built-in LED strip, so the image brightness, color, etc. are also not affected by environment light. Meanwhile, tactile images contain rich physical information such as object geometric features, contact force, contact deformation, and displacement. Based on this information, the system can achieve more precise contact control. Therefore, tactile feedback can be an alternative sensing modality for grasp pose estimation. Recent work uses tactile images from vision-based tactile sensors such as GelSight (Yuan et al., 2017), DIGIT (Lambeta et al., 2020) and GelSlim3.0 (Taylor et al., 2022) to estimate the relative pose and 3D motion field between grasped objects and tactile grippers. Meanwhile, many new types of tactile fingers (DexiTac (Lu et al., 2024)) and tactile sensors (Evetac (Funk et al., 2024)) are being applied in robotic operations. Li et al. (Li et al., 2014) use Gelsight sensors, BRISK features and RANSAC to estimate grasp pose. Gelsight produces high-quality 3D tactile images and can determine depth imprint, which improves feature detection by isolating the object from the background. DIGIT, a more affordable tactile sensor, provides a 2D RGB image but not the light incident direction (to generate the depth image). Liu et al. (2024) develops a method to reconstruct 3-D tactile motion field in real-time, that can provide rich tactile information (such as contact force) and serve as the foundation for many downstream tasks. Kelestemur et al. (2022) generates tactile image data in simulation for pose estimation of bottle caps but simulating contact and physical interaction between tactile sensors and objects with more intricate geometry is still challenging (Wang et al., 2021). In this work, we combine tactile images from a real-world PLC terminal with reinforcement learning process as part of observation. By means of contact tactile information analysis, these images enable the policy to precisely locate the terminal base and so try to minimize the contact force needed for terminal buckling.

Most prior work on tight tolerance assembly tasks (Wen et al., 2022; Li et al., 2014; Fan et al., 2019; Florence et al., 2022; Wu et al., 2025; Lin et al., 2024) leverages a single modality, such as vision, tactile, or force-torque, limiting the accuracy of the system due to occlusion, perspective effect, and sensory inaccuracy. Multi-modal systems have been explored to improve the robustness of automated insertion. Spector and Di Castro (2021), Spector et al. (2022) use RGB cameras and a force-torque sensor for learning contact and impedance control. Chaudhury et al. (2022) couple vision and tactile data to perform localization and pose estimation, and demonstrate that vision helps with disambiguating tactile signals for objects without distinctive features. Ichiwara et al. (2022) leverage tactile and vision for deformable bag manipulation by performing auto-regressive prediction. Hansen et al. (Hansen et al., 2022) use a contact-gated tactile, vision and proprioceptive observation to train reinforcement learning policies. Okumura et al. (2022) also tackle the problem of grasp pose uncertainty for insertion by using Newtonian Variational Autoencoders to combine camera observations and tactile images. Hao et al. (2025), Zhao et al. (2024) and Zhang et al. (2025) combined tactile information with large language models, achieving robotic arm manipulation of articulated objects and preference learning for insertion manipulation, respectively. They demonstrate results for USB insertion accounting for grasp pose uncertainty in one translation direction. In this work, we address terminal grasping, path planning, and terminal buckling as the whole reinforcement learning task. As the observation for the RL policy, combine two wrist camera images, one side camera image, and two tactile gripper images into visual-tactile multi-modal information. Concurrent with this was an artificial intervention program designed to guarantee a safe exploration for the policy. Our policy is able to handle both grasp pose rotation and translation uncertainty for the PLC terminal’s assembly.

## 3 Problem statement and preliminaries

**FIGURE 1:**
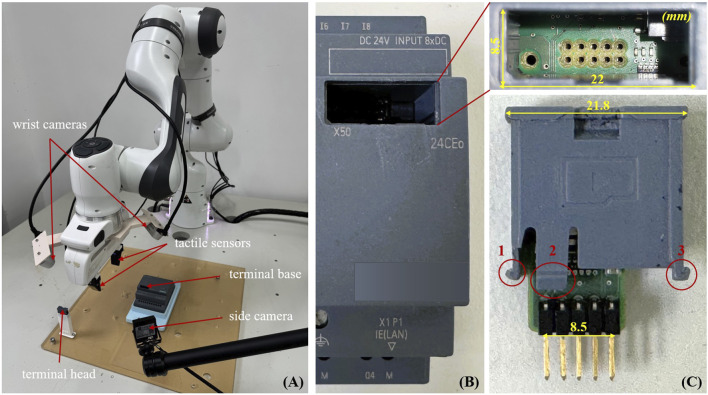
**(A)** An overview of the terminal assembly task is shown in the figure. The goal is to grasp the terminal head from the placement tray and guide the robot to the terminal base. Two RGBD cameras on the wrist and one RGB side camera are used to observe the environment. The final step is to insert the terminal head clamped by the tactile sensors onto the terminal base using visual-tactile feedback. **(B,C)** Exhibition of the components and specifics of each segment of the terminal.

Overview: We sort out a terminal assembly task for a 7-DoF robot with a parallel-jaw gripper and tactile sensors on both jaws. The end-effector has two wrist-mounted RGBD cameras, and one RGB side-camera is configured to capture the entire assembly scenario. The objective is to learn a policy that can robustly insert the terminal head into the terminal base with an unknown part’s pose within the gripper, while minimizing head-base collisions by human guidance during training. Figure 1A shows the experiment setup.

Details of the assembled terminal: As seen in Figures 1B,C, our work accomplished the PLC terminal assembly. The terminal base and the terminal head are the two components that make up the hardware. Three barbed elastic latches and ten parallel-positioned pins make up the major mating components of the terminal head. The terminal base mating area is partially enlarged in the upper right corner, where the base’s inner wall has three guide grooves that match the three spring clips, and the base’s bottom has insertion holes that match the pins. The main challenge of this work is correctly inserting the pins into the holes and snapping the three spring clips into their respective guiding grooves without causing any damage to the pins, such as bending or breaking them. Therefore, we use tactile sensing and manual intervention to minimize collision forces during the assembly process to ensure the safety of the terminal hardware.

Robotic Reinforcement Learning: Robotic reinforcement learning tasks can be defined via an Markov Decision Process (MDP) M={S,A,ρ,P,r,γ} , where s∈S is the state observation (e.g., the combination of the current environmental image, tactile image, and end-effector position), a∈A is the action (e.g., the desired end-effector pose), $\rho \left(s_{0}\right)$ is a distribution over initial states, P is the unknown and potentially stochastic transition probabilities that depend on the system dynamics, and r:S×A→R is the reward function, which encodes the task. An optimal policy π is one that maximizes the cumulative expected value of the reward, i.e., $E\left[\sum_{t=0}^{\infty }\gamma^{t}r\left(s_{t},a_{t}\right)\right]$ , where the expectation is taken with respect to the initial state distribution, transition probabilities, and policy π .

While the specification of the RL task is concise and simple, turning real-world robotic learning problems into RL problems requires care. First, the sample efficiency of the algorithm for learning π is paramount: when the learning must take place in the real world, every minute and hour of training comes at a cost. Sample efficiency can be improved by using effective off-policy RL algorithms (Konda and Tsitsiklis, 1999; Haarnoja et al., 2018; Fujimoto et al., 2018), but it can also be accelerated by incorporating prior data and demonstrations (Rajeswaran et al., 2017; Ball et al., 2023; Nair et al., 2020), which is important to achieve the fastest training times. Beyond π optimization, robotic RL has to figure out reward functions from image observations and automate initial state resets. Particularly in contact-rich tasks, the controller layer interfaces MDP actions to low-level robot controllers, necessitating safety and precision so that the RL algorithm can experiment with random actions during training.

## 4 Methods

**FIGURE 2:**
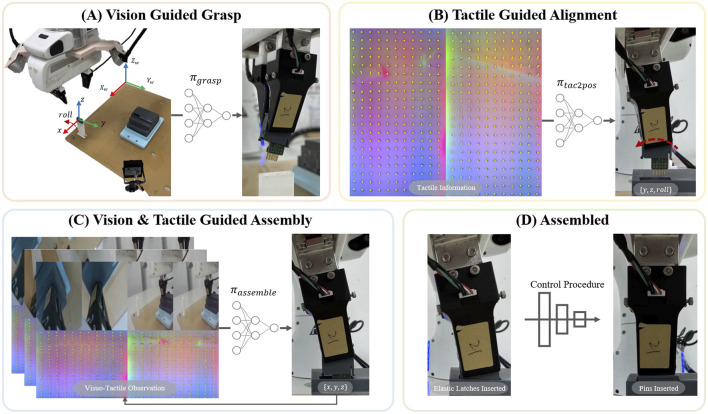
Overview of the learned three-phase assembly policy: **(A)** The vision guided policy $\pi_{\mathrm{grasp}}$ estimates the position of the terminal head and grasps it at an initial pose. **(B)** The tactile guided policy $\pi_{\mathrm{tac2pos}}$ estimates the grasp pose using the tactile image and aligns the z-axis of the terminal head with the insertion axis. **(C)** A vision-tactile multi-modal guided policy $\pi_{\mathrm{assemble}}$ is used to assemble the terminal head and the terminal base. **(D)** Following the insertion of the elastic latches, a specific procedure is executed to insert the pins, and ultimately, the entire terminal assembly is successfully completed.

In this section, we introduce our visuo-tactile feedback policies with the assistance of human intervention to address the terminal assembly problem. The overview of our method is shown in Figure 2.

### 4.1 Real-world RL for terminal grasp and assembly

#### 4.1.1 Fundamental RL algorithm

For the reinforcement learning method to be used in terminal assembly, there are two requirements: It must be (1) effective and capable of making several gradient adjustments in a time step, and (2) readily integrate prior data and then get improved with further training. In pursuit of this objective, we expand upon the recently proposed RLPD algorithm (Ball et al., 2023), which has demonstrated remarkable outcomes in sample-efficient robotic learning. The off-policy actor-critic reinforcement learning algorithm, known as RLPD, relies on the success of temporal difference algorithms with soft-actor critic (Haarnoja et al., 2018), it undergoes some significant changes to satisfy the requirements above. Three main improvements are made by RLPD: (i) high update-to-data ratio training (UTD); (ii) symmetric sampling between on-policy and prior data, where half of each batch comes from the online replay buffer and half from prior data; and (iii) layer-norm regularization during training. In order to accelerate learning, this technique can either start from scratch or leverage prior data (e.g., demonstrations). Each step of the algorithm updates the parameters of a parametric Q-function $Q_{\phi }\left(s,a\right)$ and actor $\pi_{\theta }\left(a|s\right)$ according to the gradient of their respective loss functions (Equations 1, 2):

$$L_{Q}\left(\phi \right)=E_{s,a,s^{\prime }}\left[{\left(Q_{\phi }\left(s,a\right)-\left(r\left(s,a\right)+\gamma E_{a^{\prime }∼\pi_{\theta }}\left[Q_{\bar{\phi }}\left(s^{\prime },a^{\prime }\right)\right]\right)\right)}^{2}\right]$$ (1)

$$L_{\pi }\left(\theta \right)=-E_{s}\left[E_{a∼\pi_{\theta }\left(a\right)}\left[Q_{\phi }\left(s,a\right)\right]+\alpha H\pi_{\theta }\left(\cdot |s\right)\right]$$ (2)

where $Q_{\bar{\phi }}$ is a target network, and the actor loss uses entropy regularization with an adaptively adjusted weight α . Every update step employs a sample-based approximation of each expectation, with half of the samples receiving from the replay buffer and the other half from the prior data (e.g., demonstrations). For efficient learning, multiple update steps are performed per time step in the environment, which is referred to as the update-to-date (UTD) ratio. Regularizing the critic with layer normalization enables higher UTD ratios and more effective training (Ball et al., 2023).

In this work, $\pi_{\mathrm{grasp}}$ and $\pi_{\mathrm{assemble}}$ are trained based on RLPD. And the three improvements of RLPD have also demonstrated their advantages in handling task-specific challenges in our experiments: (i) High UTD ratio: Our training shows that a UTD ratio of 20 reduced wall-clock training time by 47% compared to a UTD ratio of 5 (a common baseline in off-policy RL). This acceleration is critical for real-world assembly, where hardware access is constrained; (ii) Symmetric sampling: Replaying training data (without modifying hardware interactions) revealed that removing symmetric sampling (using 100% online data) increased Q-function loss variance by 63%—indicating unstable learning from contact-driven data fluctuations. In contrast, symmetric sampling maintained loss variance ≤ 5% across epochs; (iii) Layer normalization: Omitting layer normalization caused the policy to diverge in 32% of training trials (vs. 0% with normalization), as it failed to adapt to sudden tactile signal shifts (e.g., from no contact to hard contact with the terminal base).

#### 4.1.2 Classifier-based reward specification

Reward functions are difficult to specify by hand when learning with image observations, as the robot typically requires some sort of perception system just to determine if the task was performed successfully. While some tasks can accommodate hand-specified rewards based on the location of the end effector (under the assumption that the object is held rigidly in the gripper), most tasks require rewards to be deduced from images. In this case, the reward function can be provided by a binary classifier that takes in the state observation s and outputs the probability of a binary “event” e , corresponding to successful completion. The reward is then given by r(s)=log⁡p(e|s) .

This classifier can be trained either using hand specified positive and negative examples, or via an adversarial method called VICE (Fu et al., 2018). The latter addresses a reward exploitation problem that can arise when learning with classifier based rewards, and removes the need for negative examples in the classifier training set: when the RL algorithm optimizes the reward r(s)=log⁡p(e|s) , it can potentially discover “adversarial” states that fool the classifier p(e|s) to erroneously output high probabilities. VICE addresses this issue by adding all states visited by the policy into the training set for the classifier with negative labels, and updating the classifier after each iteration. In this way, the RL process is analogous to a generative adversarial network (GAN), with the policy acting as the generator and the reward classifier acting as the discriminator. We trained corresponding classifiers for $\pi_{\mathrm{grasp}}$ and $\pi_{\mathrm{assemble}}$ in this work. Set the visual image observation for $\pi_{\mathrm{grasp}}$ as a positive example $\left(N_{p}=200\right)$ when the gripper successfully grabs the terminal head in the initial bin, and the others as negative examples $\left(N_{n}=800\right)$ . In the case of $\pi_{\mathrm{assemble}}$ , the visual-tactile multi-modal observation is set as a positive example $\left(N_{p}=600\right)$ when the terminal head’s elastic latches gets inserted into the terminal base; the other situations are set as negative examples $\left(N_{n}=2400\right)$ .The rationale for the $N_{p}:N_{n}=1:4$ ratio lies in the fact that, through our repeated experiments, classifiers trained on datasets adhering to this ratio yield the highest classification accuracy.

#### 4.1.3 Actor and learner nodes

**FIGURE 3:**
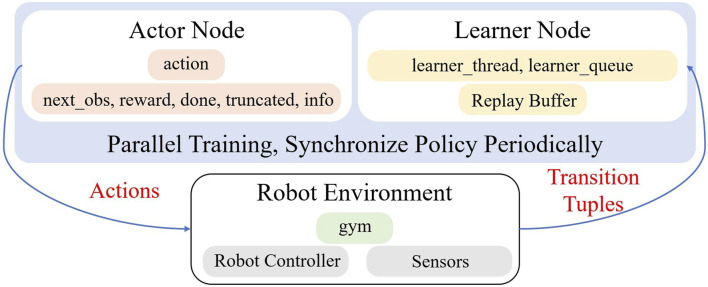
Policy training and real-world robot architecture. Three parallel processes, consisting of the actor, which chooses actions, and the learner node, which actually runs the training code, and the robot environment, which executes the actions from the actor and contributes data back to the learner.

In order to decouple inferring actions and updating policies, this work incorporates alternatives for training and acting in tandem, as seen in Figure 3. In sample-efficient real-world learning tasks with large UTD ratios, we discovered that this was advantageous. Our policy reduces the overall wall-clock time spent training in the real world while maintaining the control frequency at a fixed rate, which is essential for tasks requiring instant feedback and reactions, like deformable objects and contact-rich manipulations (e.g., terminal assembly). This is achieved by separating the actor and learner on two separate threads.

### 4.2 Supervised learning for tactile guided alignment

Data Collection: The terminal head fixed in the initial bin throughout data collection. We explore grasp pose variations in 3-DoF ( y,z translation and x -axis rotation roll , Figure 2A left). We perform uniform random sampling over the range [−6,6]mm , [−7,3]mm , $\left[-\frac{\pi }{6},\frac{\pi }{6}\right]rad$ for y,z,roll , with 12, 10 and 60 samples respectively. The robot closes the gripper with a force of 50N at each of the sampled poses and records the pair of tactile image readings and y,z,roll . We collect 7,200 pairs of tactile images ( 700×400 pixels, RGB) by Xense G1-WS vision-based tactile sensor as data points in 300 min.

Alignment Policy: We adopted RegNet 3.2 GF (Radosavovic et al., 2020) as the backbone of the policy network and replaced its last layer with a linear layer producing 3 outputs. Using the aforementioned data—comprising pairs of tactile images (Figure 2B, 700×800 pixels, RGB) corresponding to grasp poses of the PLC terminal (Figure 1C)—we trained an alignment policy $\pi_{\mathrm{tac2pos}}$ that outputs the desired End-Effector displacement (y,z,roll) to align the terminal head with the terminal base (Figure 2B) given a tactile image. Tactile image augmentation was performed by randomly jittering brightness and contrast within the range U[0.8,1.2] ; the jitter range settings were influenced to a certain extent by the geometric features of the grasped terminal head. Regarding hyperparameters, we used a batch size of 128, an initial learning rate of 1e-3 with a decay factor of 0.99 every 100 gradient steps, mean squared error as the loss function, and the Adam optimizer (Kingma, 2014). These hyperparameters represent optimal values determined through multiple experiments based on the collected raw data and are task-adaptable rather than universal, requiring further adjustment when using different tactile sensors or grasping different objects in future work.

### 4.3 Impedance controller for terminal assembly

During the experiment, we found that the choice of controllers can heavily affect the final performance. This is more pronounced for contact-rich manipulation. In this work (Figures 2C,D), an overly stiff controller might bend the fragile pins and make insertion difficult, while an overly compliant controller might struggle to move the object into position quickly.

A typical setup for robotic RL employs a two-layered control hierarchy, where an RL policy produces setpoint actions at a much lower frequency than the downstream real-time controller. The RL controller can set targets for the low-level controller, but such targets may lead to physically undesirable consequences—especially in contact-rich manipulation tasks—if not regulated by a robust low-level control mechanism. To this end, the impedance controller is integrated into this hierarchy as a core component, with its framework encompassing a spring-damper-based force objective and a critical error-bounding safety constraint. A typical impedance control objective for this controller (Equation 3) is

$$F=k_{p}\cdot e+k_{d}\cdot \dot{e}+F_{ff}+F_{\mathrm{cor}}$$ (3)

where $e=p-p_{\mathrm{ref}}$ , p is the measured pose of the end-effector, and $p_{\mathrm{ref}}$ is the target pose computed by the upstream controller. Here, $F_{ff}$ is the feed-forward force (used to compensate for static loads like gravity), and $F_{\mathrm{cor}}$ is the Coriolis force (to mitigate dynamic disturbances from robot motion). This force objective is then converted into joint space torques by multiplying with the Jacobian transpose, offset by nullspace torques to maintain stable joint behavior. By design, the controller acts as a spring-damper system around the equilibrium set by $p_{\mathrm{ref}}$ : $k_{p}$ (stiffness coefficient) governs the response to position deviations, while $k_{d}$ (damping coefficient) smooths motion to avoid oscillations. As described above, this system will yield large forces if $p_{\mathrm{ref}}$ is far away from the current pose, which can lead to a hard collision or damage when the arm is in contact with objects (e.g., during PCB insertion). Therefore, it’s crucial to constrain the interaction force generated by it. However, directly reducing $k_{p}$ or $k_{d}$ will hurt the controller’s positional accuracy. Thus, we bound e so that |e|≤Δ (a predefined safety threshold), and the generated force from the spring-damper system will be bounded to $k_{p}\cdot |\Delta |+2k_{d}\cdot |\Delta |\cdot f$ , where f is the control frequency of the low-level controller. This error-bounding step completes the impedance controller framework, ensuring it balances precision and safety for real-world robotic RL tasks.

## 5 Experiments

In this section, we introduce the experimental setup of the assembly task and the evaluation of the proposed methods.

### 5.1 Experiment setup

We consider a terminal assembly task using a Franka Emika Panda Robot (7-DoF), equipped with a parallel-jaw gripper with XENSE G1-WS vision-based tactile sensors (used in AgiBot World Colosseo (Bu et al., 2025)) mounted on both jaws. The G1-WS sensor, independently developed by our laboratory, captures RGB tactile images with a fixed resolution of 700 × 400 pixels—matching the sampling resolution of commercial GelSight (Yuan et al., 2017) mini sensors—and offers advantages including a lower cost ($300) compared to GelSight mini ($500), a larger sensing area (17.5 (H) × 29.5 (V) mm) than GelSight mini (18.6 (H) × 14.3 (V) mm), and a wedge-shaped structure that adapts to diverse assembly environments. For the alignment policy training (4.2), paired tactile images from both gripper jaws were concatenated horizontally to form a single 700 × 800 pixel input, ensuring simultaneous capture of contact information from both sides of the terminal head.

The end effector is equipped with two wrist-mounted Intel RealSense Depth Camera D435i RGBD cameras, selected for their high-quality 1,280 × 720 RGB imaging at up to 90 fps—ensuring clear, temporally consistent visual data for dynamic manipulation scenarios. Time synchronization between visual and tactile data was achieved via two steps: (1) Hardware triggering: The D435i cameras and G1-WS tactile sensors were connected to a common GPIO trigger module, ensuring all sensors initiate sampling within a 1 ms time window; (2) Software timestamping: Each sensor frame (visual/tactile) was tagged with a high-precision system timestamp (resolution: 100 μ s) via Robot Operating System (ROS) topics. The D435i’s 90 fps sampling frequency was downsampled to 30 fps (matching the G1-WS’s 30 Hz rate) by selecting the visual frame with the timestamp closest to each tactile frame—resulting in a maximum synchronization error of <5 ms, which is negligible for terminal assembly tasks. This setup guarantees consistency between multi-modal observations.

The D435i′s compact form factor minimizes interference with the gripper and assembly components, while its robust SDK (compatible with ROS and Python) facilitates seamless integration into our custom control pipeline. It also delivers reliable performance under varying lighting conditions, including low-light environments, ensuring stable data quality throughout experiments. Additionally, a jieruiweitong DF100 RGB side-camera is configured to capture the entire assembly scene (Figure 1), chosen for its 1,280 × 720 resolution, 30 Hz sampling rate, and cost-effectiveness ($20).

At the beginning of each training and evaluation episode, the initial end effector pose is sampled uniformly (N=100) from a starting region Ω : $x\in \left[-3,3\right]cm,y\in \left[-3,3\right]cm,z\in \left[-5,3\right]cm,roll\in \left[-\frac{\pi }{6},\frac{\pi }{6}\right]rad$ . Meanwhile, we initialize RL training from 30 teleoperated demonstrations (Section 4.1.1) using a Joystick (BTP-A1N3S). All training was done on a single Nvidia RTX 4090 GPU.

### 5.2 Experimental procedure

At the beginning of each test experiment, the end effector is set to the initial pose sampled from Ω (Figure 2A left). From this starting pose, the robot first executes the grasp policy $\pi_{\mathrm{grasp}}$ to visuoservo and grasps the terminal head—leveraging RGB-D data from the D435i cameras for precise localization of the terminal head in the initial bin. During the removal of the terminal head, minor jitter introduced by $\pi_{\mathrm{grasp}}$ may lead to a collision between the terminal head and the initial bin, thereby causing an error in the grasping posture. Specifically, the gripper remains vertically aligned downward, whereas the terminal head exhibits misalignment with the receptacle in both translational and rotational dimensions (Figure 2A right).

Then the robot activates the align policy $\pi_{\mathrm{tac2pos}}$ , which processes tactile images from the G1-WS sensors to estimate the terminal head’s relative pose (y/z translation and roll rotation) and outputs corrective movements to align the terminal head’s insertion axis with the terminal base (Figure 2B). The G1-WS’s large sensing area and high-resolution imaging ensure accurate pose estimation, while its wedge-shaped design avoids interference with the gripper during alignment.

After the alignment, the vision-tactile guided assembly policy $\pi_{\mathrm{assemble}}$ is executed to insert the elastic latches (Figure 2C), fusing D435i visual data (for environmental context) and G1-WS tactile feedback (for contact detection). Due to the structural redundancy and ductility of the assembled PLC terminal, once all elastic latches are properly inserted, a simple vertical downward force applied to the terminal head is sufficient to ensure complete insertion of all pins. Accordingly, we developed an open-loop control program to execute the final pin insertion process (Figure 2D). The robot then resets to the next initial sampled pose, waiting for the next test.

During the policy training and testing process, human intervention was triggered by a hybrid mechanism combining manual visual observation and automatic force sensing, with clearly defined termination conditions: (i) Successful termination: The robot successfully grasps the terminal head (grasp binary classifier output=1) and completes the assembly after adjusting the grasping pose (assemble binary classifier output=1) . (ii) Grasp failure intervention: Triggered when the grasp binary classifier output=0 for 5 consecutive seconds (indicating unstable grasp). Intervention was initiated via Joystick by the experimenter to manually re-grasp until the grasp binary classifier output=1 , after which the task terminates. (iii) Deviation/collision intervention: Triggered by two complementary cues: (a) Manual visual observation: the experimenter initiated intervention upon visually detecting the terminal head deviating from the terminal base or colliding with non-target components; (b) Automatic force sensing: The system automatically paused motion and prompted intervention if the EE force-torque sensor detected a collision force ≥ 30 N. Upon intervention, the experimenter manually completed assembly until the assemble binary classifier output=1 , then the task terminates. Notably, in both conditions (ii) and (iii), the data collected during manual intervention is stored as expert demonstration data into the replay buffers of $\pi_{\mathrm{grasp}}$ and $\pi_{\mathrm{assemble}}$ respectively, to guide and accelerate policy training.

### 5.3 Comparison and ablation studies

Examine the function and significance of the RLPD algorithm: As outlined in Section 4.1.1, the most distinctive characteristic of the RLPD algorithm lies in its integration of human prior demonstrations to guide the learning process, which effectively reduces both training time and sample complexity. To assess the necessity of these demonstrations, we compare our approach with the Twin Delayed Deep Deterministic Policy Gradient (TD3), an off-policy Actor-Critic algorithm derived from DDPG. TD3 belongs to the class of online reinforcement learning algorithms that require continuous interaction with the environment and rely solely on trial-and-error learning to discover optimal policies, without incorporating human demonstrations. The comparison is conducted under identical environmental settings: (1) Exploration noise: Gaussian noise with standard deviation = 0.1 (applied to end-effector pose commands); (2) Learning rate: 1e-3 for actor/critic networks (Adam optimizer); (3) Training epochs: 200 epochs (1,000 steps per epoch); (4) Network architecture: Same 3-layer actor/critic structure (consistent with RLPD’s base design).

Furthermore, to demonstrate that expert demonstrations alone are insufficient for task completion, we also evaluate a behavioral cloning (BC) baseline trained on 150 high-quality expert teleoperated demonstrations. This dataset size approximately matches the total amount of data stored in the RLPD replay buffer at convergence. To ensure fair comparison: (1) Network architecture: BC used the same RegNet 3.2 GF backbone as RLPD’s alignment policy $\left(\pi_{\mathrm{tac2pos}}\right)$ , with an output layer predicting end-effector poses; (2) Training epochs: 200 epochs (matching RLPD), batch size = 128. It is important to note that this BC baseline utilizes five times more demonstration data than the number of demonstrations required by our method. Meanwhile, to intuitively verify the role of “human prior demonstrations” in the RLPD algorithm, we replaced the demo buffer with a subset of replay buffer data in one training session to isolate and examine the function of human demonstrations.

**FIGURE 4:**
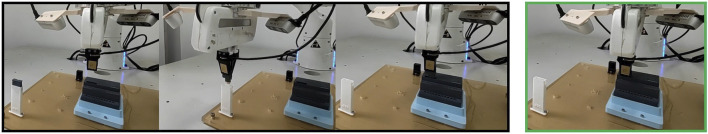
Illustration of the robot performing terminal assembly with our method. The green box indicates a state where the robot receives classifier reward for completing the task.

We report the results in Table 1, and show example executions in Figure 4. Training the TD3 policy in the physical environment resulted in divergence across all conducted training trials. In each case, the terminal head collided with the terminal base during the training of $\pi_{\mathrm{assemble}}$ , causing significant changes to the relative grasp pose or inflicting damage to the pins and the tactile sensor gel pad. Such issues cannot be directly corrected due to the absence of a reliable recovery procedure that can systematically restore the grasp pose without human demonstrations. Our policies significantly outperform BC baselines, even when trained with five times fewer demonstrations than BC. This indicates that relying solely on demonstrations is insufficient for achieving optimal performance. In addition to achieving up to a tenfold improvement in success rate over BC methods, our approach also reduces training time by up to twofold. Removing real-time human intervention data from the buffer leads to a 68% drop in success rate (from 100 to 32), confirming the buffer’s role in addressing rare failure modes (Table 1, RLPD (w/o demo)). We also observed from the aforementioned experiments that the terminal head rotation and translation estimated based on tactile images $\left(\pi_{\mathrm{tac2pos}}\right)$ exhibit a high degree of accuracy (see Table 2).

**TABLE 1:** Results suggest that (1) frequent slippage and rotations of the terminal head caused by collisions with the terminal base lead to failure in training TD3, (2) the BC trained solely on 150 human demonstrations is insufficient for training an accurate assembly model and (3) the human demonstrations play an important role in improving training efficiency and policy success rate. Our approach outperforms both baseline policies.

Algorithms	# Of demos	Env input	Training time	Success/Total
TD3	0	Yes	285 min	0/100
BC	150	No	105 min	9/100
RLPD (w/o demo)	0	Yes	265 min	32/100
RLPD (Ours)	30	Yes	55 min	100/100

**TABLE 2:** Mean and standard deviation of the error in estimating the relative grasp pose (y,z,roll) of the terminal head using the tactile-based pose estimation policy $\pi_{\mathrm{tac2pos}}$ , evaluated over 100 sampled initial end effector poses.

Error Dimension	y (mm)	z (mm)	roll (rad)
Mean Error	8.63e-2	1.28e-1	5.76e-3
Standard Deviation	4.28e-3	6.13e-2	4.23e-3
Success Threshold (ME)	1.50e-1	2.00e-1	1.80e-2

Exploring Utility of Tactile and Vision Information: We perform study the relative benefits of using tactile and vision for assembly term $\left(\pi_{\mathrm{assemble}}\right)$ . We test 3 different approaches: (1) A Tactile Only approach (Figure 2C, the lower part of Visuo-Tactile Observation) (2) A Vision Only approach (Figure 2C, the upper part of Visuo-Tactile Observation) and (3) a Combined Approach (Ours). We perform experiments with the three different approaches with the same procedure as in Section 5.2 and report results in Table 3.

**TABLE 3:** Ablation study with comparing single modal Tactile Only, Vision Only, and a Combined two-modal approach leveraging tactile and visual information.

Observation	Training time	Success/Total
Tactile Only	195 min	23/100
Vision Only	60 min	1/100
Vision + Tactile	55 min	100/100

The Tactile Only model achieved successful assembly 23/100 times. However, its training time exceeded 3 times that of the other two models. This is because, across much of the exploration range, no contact occurred between the terminal head and terminal base, resulting in static tactile sensor images. Consequently, a significant portion of the training process involved the policy exploring for the position of the terminal base. These findings suggest that visual observation is essential for estimating the approximate location of the terminal base, enabling the policy to actively reduce the exploration space and accelerate learning. In contrast, the Vision Only model exhibited faster convergence during training but performed poorly in completing the assembly task, achieving only one success in 100 attempts. This limitation stems from the absence of fine-grained tactile feedback regarding contact events, highlighting the necessity of tactile sensing for millimeter-level positional estimation in contact-rich tasks. The multi-modal model, which integrates both tactile and visual inputs, outperforms either modal approach by combining tactile-based terminal head position prediction with vision-based implicit estimation of environmental states. This synergy demonstrates that the integration of tactile and visual observations effectively reduces uncertainties inherent in assembly tasks.

## 6 Discussion

In conclusion, we propose an effective and safe methodology for acquiring a visuo-tactile insertion policy within real-world reinforcement learning (RL) environments characterized by unknown component positions and grasping configurations. This is achieved by leveraging human demonstrations to accelerate the training process while maintaining the safety of the components, alongside the implementation of a structured three-phase assembly framework that delineates the task into distinct stages—grasping, alignment, and insertion—facilitated by integrated tactile and visual feedback.

### 6.1 Limitations

Although our results are promising, several limitations of the proposed approach remain. First, the generalizability of our method has yet to be validated across various assembly tasks, particularly those involving objects with more intricate geometric properties (e.g., non-prismatic components with curved mating surfaces) or scenarios where the physical dimensions significantly deviate from the scale of the tactile sensor (e.g., micro-assembly tasks with parts < 5 mm or large components > 50 mm). The current tactile pose estimation policy $\pi_{\text{tac}2\text{pos}}$ is trained specifically on PLC terminals, and its performance degrades when applied to parts with distinct contact patterns (e.g., smooth metallic vs. textured plastic surfaces). Second, components composed of different materials may necessitate the application of distinct pose estimation algorithms: for example, slippery materials (e.g., Teflon-coated terminals) introduce slippage between the gripper and part, which the current tactile model does not explicitly account for. Third, during the collection of human demonstrations and the training phase, the unique characteristics of the assembled programmable logic controller (PLC) in this study require a human operator to manually detach the terminal head following each successful assembly to reset the environment. This manual intervention not only extends the training duration (adding 10s per trial) but also introduces variability due to inconsistencies in human execution (e.g., varying detachment forces that alter the initial bin’s part placement).

### 6.2 Future work

To address these limitations, future research should focus on three main directions. First, generalizing the proposed methodology to encompass assembly tasks involving objects with diverse shapes, materials, and dimensions: this will involve developing few-shot tactile pose estimation models that adapt to new parts with minimal retraining data, as well as integrating material property estimation (e.g., friction coefficient) from tactile images to handle slippage—directly addressing the need for multi-material terminal adaptation in industrial scenarios. Specifically, we aim to extend the current PLC terminal-focused framework to metallic, Teflon-coated, and composite-material terminals, where varying surface properties (e.g., friction coefficients ranging from 0.2 to 0.6) require adaptive tactile signal interpretation and grasp force adjustment. Second, the development of an automated reset learning framework tailored specifically for terminal insertion and extraction processes: this framework could leverage the existing $\pi_{\text{tac}2\text{pos}}$ policy to detect successful assembly, followed by a learned “extraction policy” that uses tactile feedback to safely detach the terminal head without human intervention—significantly improving the efficiency and reliability of such systems. Concurrently, we will investigate batch assembly efficiency optimization by integrating real-time sensor drift compensation (e.g., calibrating tactile image brightness and depth accuracy across 100+ consecutive assembly cycles) and adaptive RL policy updates to mitigate performance fluctuations induced by environmental wear (e.g., gripper fatigue) or component batch variations. Third, optimizing the multi-modal policy for edge deployment: techniques such as model quantization and knowledge distillation will be explored to reduce the computational footprint of the RegNet backbone and RLPD-based policy, enabling real-time inference on embedded GPUs. Additionally, future work will investigate the integration of foundation models for visual-tactile fusion, which could eliminate the need for task-specific classifiers by leveraging pre-trained knowledge of object interactions. Finally, validating the method in industrial factory settings with variable lighting, vibration, and part tolerances will be critical to demonstrating its practical applicability—with a focus on validating multi-material adaptation and batch efficiency improvements in real-world production lines.

## Data Availability

The raw data supporting the conclusions of this article will be made available by the authors, without undue reservation.
